# NICE polyp feature classification for colonoscopy screening

**DOI:** 10.1007/s11548-025-03338-9

**Published:** 2025-03-13

**Authors:** Thomas De Carvalho, Rawen Kader, Patrick Brandao, Laurence B. Lovat, Peter Mountney, Danail Stoyanov

**Affiliations:** 1 Odin Vision, London, UK; 2 Department of Computer Science, UCL Hawkes Institute, University College London, London, UK; 3 Division of Surgery and Interventional Science, University College London, London, UK; 4 Gastrointestinal Services, University College London Hospital, London, UK

**Keywords:** Surgical data science, Polyp classification, Deep learning, Colonoscopy

## Abstract

**Purpose:**

Colorectal cancer is one of the most prevalent cancers worldwide, highlighting the critical need for early and accurate diagnosis to reduce patient risks. Inaccurate diagnoses not only compromise patient outcomes but also lead to increased costs and additional time burdens for clinicians. Enhancing diagnostic accuracy is essential, and this study focuses on improving the accuracy of polyp classification using the NICE classification, which evaluates three key features: colour, vessels, and surface pattern.

**Methods:**

A multiclass classifier was developed and trained to independently classify each of the three features in the NICE classification. The approach prioritizes clinically relevant features rather than relying on handcrafted or obscure deep learning features, ensuring transparency and reliability for clinical use. The classifier was trained on internal datasets and tested on both internal and public datasets.

**Results:**

The classifier successfully classified the three polyp features, achieving an accuracy of over 92% on internal datasets and exceeding 88% on a public dataset. The high classification accuracy demonstrates the system’s effectiveness in identifying the key features from the NICE classification.

**Conclusion:**

This study underscores the potential of using an independent classification approach for NICE features to enhance clinical decision-making in colorectal cancer diagnosis. The method shows promise in improving diagnostic accuracy, which could lead to better patient outcomes and more efficient clinical workflows.

## Introduction

Colorectal cancer is the third most common cancer worldwide, with nearly two million new cases reported according to the World Cancer Research Fund International. Colonoscopy is the gold standard for diagnosing colorectal cancer by detecting precancerous lesions called polyps. Identifying and removing adenomas (cancerous polyps) is crucial, as each undetected adenoma increases cancer risk and reduces patient survival chances. Research indicates that a 1% increase in adenoma detection rate correlates with a 3% reduction in cancer risk [1].

Polyps are either cancerous (adenomas) or non-cancerous (hyperplastic), requiring different approaches during colono-scopy. Adenomas should be removed, while hyperplastic polyps can often be left untreated. Accurate identification is essential to avoid unnecessary costs, time, and potential risks to the patient from incorrect diagnoses.

Three main systems classify polyps: the Paris classification, the Kudo classification, and the Narrow-Band Imaging (NBI) International Colorectal Endoscopic (NICE) classification [2]. This study focuses on the NICE classification, which categorizes polyps into three types based on vessels, surface patterns, and colour (see Table 1). Type-1 polyps are likely hyperplastic, type-2 are adenomas, and type-3 indicate deep submucosal invasive cancer.

**Table 1:**
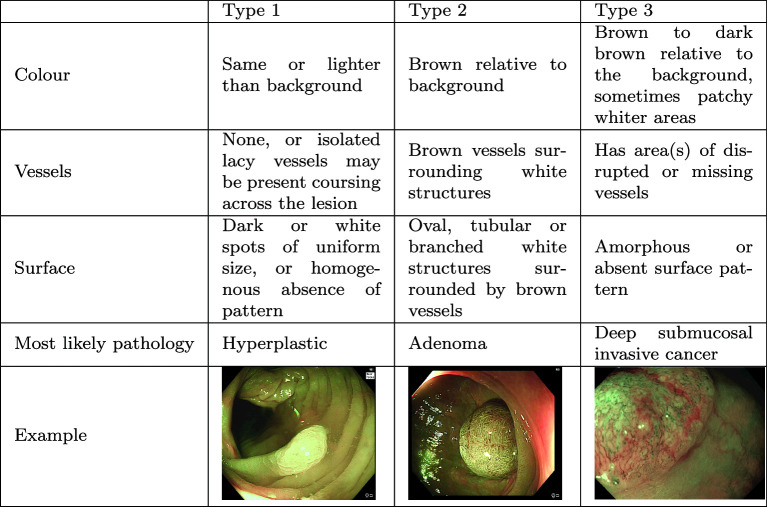
NICE classification [2]

While histology (polyp removal or biopsy) is the most accurate diagnostic method, it is expensive and time-consuming. An alternative is on-site diagnosis using NBI and the NICE classification during colonoscopy, which reduces costs and time. However, clinician effectiveness can vary significantly based on experience. Although datasets for polyp diagnosis are limited compared to those for polyp detection, private datasets have enabled numerous studies focussing on polyp classification.

Previous research on polyp classification has predominantly focussed on feature extraction using handcrafted features. Early methods employed vascularization features, texture analysis with local binary patterns, colour and texture features combined with classifiers like support vector machines (SVM), and shape-based features using curvature and Delaunay triangulation [3–9]. Wavelet transforms and discrete cosine transforms were also explored for scale-invariant features [10–12]. While these approaches have contributed to polyp classification, the reliance on handcrafted features often results in models that are not easily interpretable by clinicians.

With advancements in deep learning, convolutional neural networks (CNNs) have been increasingly applied to polyp classification tasks. Ribeiro et al. (2016) [13] pioneered this approach, followed by studies employing transfer learning, comparing CNNs with handcrafted methods, and exploring various CNN architectures [14–18]. Ensemble methods and context-sensitive models have also been proposed to improve classification accuracy [19–21]. Despite these advancements, many studies rely on private datasets and classify polyps into only a few categories, limiting direct comparisons and clinical interpretability.

This study aims to address these limitations by proposing a methodology that classifies distinct clinical features of polyps, namely vessels, surface patterns, and colour, within a unified framework. By aligning the classification with medical criteria and focussing on features interpretable by clinicians, the proposed system seeks to enhance diagnostic decision-making grounded in clinical reasoning. Integrating the analysis of all three features under a single network not only streamlines the process but also contributes to a more clinically relevant understanding of polyp characteristics. To the best of our knowledge, no prior study has specifically focussed on the classification of NICE features. This study aims to provide a framework for classifying NICE features to assist clinicians in making informed decisions, rather than directly predicting a diagnosis.

**Fig. 1:**
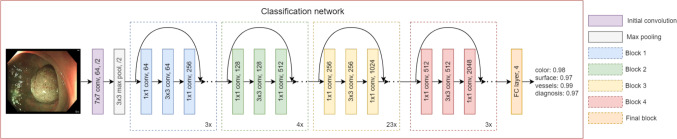
Architecture of the networks used for the classification task of the polyp features

## Methods

The objective is to classify the texture, colour, and vessel features of a polyp according to the NICE classification using a deep neural network based on ResNet [22]. The third category of the NICE classification pertains to deep submucosal invasive cancer, which is rare and relatively straightforward for doctors to classify. Due to limited data, this study focuses on the first two categories: adenoma and hyperplastic polyps. This creates a multiclass problem with two labels for each feature category. The diagnosis is also predicted during the training process, as the label is already available and provides additional valuable information to enhance the model’s learning and overall accuracy. The model employed is a ResNet-101, pretrained on ImageNet, with four classes, as depicted in Fig. 1.

The model classifies four features of polyps: colour, surface pattern, vessels, and diagnosis. Diagnosis prediction is utilized solely during the training phase, as the primary objective of this study is to provide clinicians with detailed information on NICE features to support their diagnostic decision-making, rather than offering a direct diagnosis. Initially, binary cross-entropy loss with sigmoid activation is used because each class is independent, allowing for separate predictions.

To handle low-quality frames that cannot be classified as type-1 or type-2—due to issues like camera movement or light reflection, an “indistinguishable” label is introduced. This addition transforms the problem into a multiclass, multi-label classification with three mutually exclusive labels for each feature. Examples of images with the “indistinguishable” label are shown in Table 2.

**Table 2:**
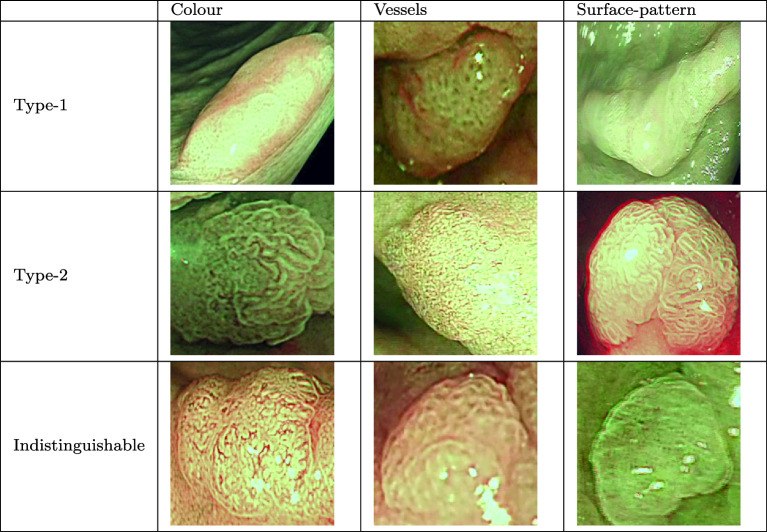
Examples of type-1, type-2, and indistinguishable images for each class

**Table 3:** Overview of the datasets used in this study

	Status	Patients	Adenomas	Non-adenomas
Dataset 1 (Full procedures)	Private	391	262	140
Dataset 2 (Clips)	Private	573	596	293
PICCOLO	Public	40	50	20

**Table 4:** Summary of the number of frames corresponding to each label and feature utilized in this study

		Dataset 1 (Full procedures)	Dataset 2 (Clips)	PICCOLO
	Type-1	26821	476	739
Colour	Type-2	50740	979	2235
	Indistinguishable	3211	608	66
	Type-1	27517	472	721
Vessels	Type-2	51407	921	2233
	Indistinguishable	1848	670	86
	Type-1	26038	471	754
Surface-pattern	Type-2	52519	910	2208
	Indistinguishable	2215	682	78
Diagnosis	Non-adenoma	23293	868	757
	Adenoma	57482	1811	2526

**Fig. 2:**
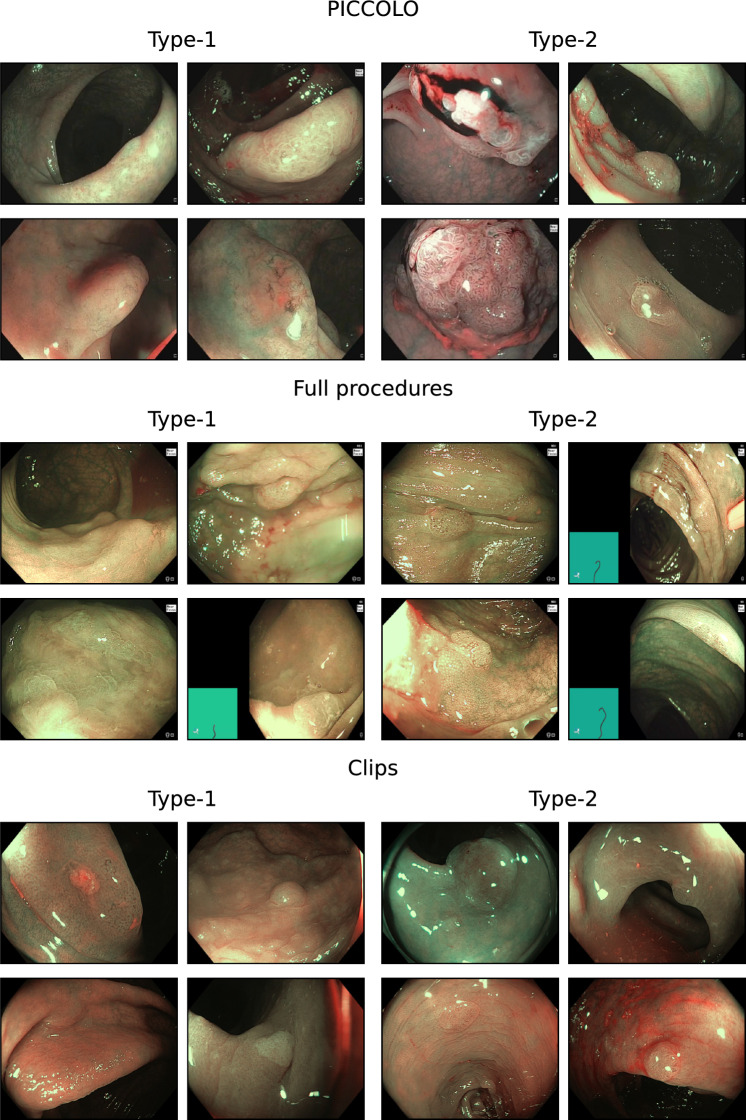
Examples of images from each dataset, with type-1 polyps on the left and type-2 polyps on the right. Some polyps can be challenging to locate. All annotated frames provide a clear view of the polyp and utilize the NBI modality to ensure diagnostic accuracy

With the inclusion of the “indistinguishable” label, the loss function changes to standard cross-entropy loss, and the activation function switches to softmax to ensure only one label is predicted per feature. The final loss is a combination of losses from each feature: colour, vessels, surface pattern, and diagnosis. The final loss $L_{\text {final}}$ is defined as:

1 $$\begin{aligned} L_{\text {final}} = L_{\text {colour}} + L_{\text {vessels}} + L_{\text {surface}} + L_{\text {diagnosis}} \end{aligned}$$

The dataset is split into 60% for training, 10% for validation, and 30% for testing, ensuring a proportional representation of each feature through k-means clustering and stratified sampling. Metrics used for clustering include the number of polyps, adenomas, non-adenomas, Narrow-Band Imaging (NBI) frames, and high-quality frames. During training, classes are balanced to maintain equal numbers of adenomas and non-adenomas.

Frames extracted from colonoscopy videos are resized to 224$\times$224 pixels and normalized using ImageNet’s pixel distribution values. The model is trained with a learning rate of 0.0001, a batch size of 64, and the Adagrad optimizer [23]. On the fly data augmentation techniques such as rotation ($-180$°  to   180°), translation ($-20$% to 20%), scaling (0.8 to 1.2), shearing ($-20$°  to   20°), and colour adjustments (brightness: 0–0.5, contrast: 0–0.5, saturation: 0–0.5) are applied to enhance model robustness. The model outputs four predictions—one for each class—with each prediction representing the probability of the feature being type-2.

As a final processing step, thresholding is applied to all predictions. Let *t* be the threshold and *p* the raw prediction output by the model. The final prediction, $p_f$, is then defined as follows:

2 $$\begin{aligned} p_f= {\left\{ \begin{array}{ll} 1 &  \quad \text {if } \ p > 0.5 + t\\ 0 &  \quad \text {if} \ p< 0.5 - t\\ N/A &  \quad \text {if} \ 0.5 - t< p < 0.5 + t \end{array}\right. } \end{aligned}$$

In this context, “N/A” signifies that the prediction is discarded due to insufficient confidence to ensure reliability. For instance, with a threshold of 0.2, a prediction of above 0.7 would result in a type-2 classification, while a prediction below 0.3 would be type-1. All the other predictions would be discarded. This thresholding step effectively filters out uncertain cases, focussing on retaining only the most accurate predictions. The model checkpoint with the highest validation accuracy is selected for final evaluation.

## Results and discussion

### Datasets

No public dataset with annotations for the NICE features is available. Therefore, three datasets were annotated by a gastroenterology doctor with expertise in artificial intelligence applied to colonoscopy. An overview of the three datasets is provided in Tables 3, 4, and in Fig. 2. Only high-quality NBI frames were annotated since the NICE classification is specific to the NBI modality, and low-quality frames render diagnosis impossible. Each frame is annotated with four independent labels, corresponding to the classes: colour, vessels, surface pattern, and diagnosis. These labels are assigned independently of one another.

The first dataset is a private dataset containing 391 videos with 402 polyps, including 262 adenomas and 140 non-adenomas. Data were collected using an Olympus 290 scope at the University College London Hospital. The second dataset is another private dataset collected with an Olympus 190 scope, comprising 889 polyps from 573 procedures. Frames were annotated for 596 adenomas and 293 non-adenomas. The last dataset is the public PICCOLO dataset [24], which includes images from clinical colonoscopy videos collected with an Olympus 190, including NBI images. It comprises 70 different lesions from 40 patients, with 50 adenomas and 20 non-adenomas. Only the private datasets were used for training, while PICCOLO was used solely for testing.

**Table 5:** Results of the classification network on a subset of the full procedures dataset

	A		B	
	Sensitivity (%)	Specificity (%)	Sensitivity (%)	Specificity (%)
*Full procedures*				
Colour	96.1	91.9	**96**.**8**	**92**.**0**
Surface	98.7	**86**.**5**	**99**.**2**	85.4
Vessels	98.5	**91**.**9**	**98**.**6**	84.1

Model A is trained without indistinguishable labels, and model B is trained with them. The best performance for a given metric and feature between models A and B is highlighted in bold

These datasets initially contained either detection or segmentation labels for polyps, where the label could be a detailed segmentation mask or a bounding box around the polyp. The annotations were then expanded to include labels for each feature: type-1, type-2, or indistinguishable. Type-1 and type-2 correspond to the NICE classification, while the indistinguishable label is applied when artefacts like light reflections make feature classification impossible. This label is also used for frames that are out of focus or blurry due to camera movements, as shown in Table 2. During the training and testing phases, images are cropped to include only the content within the detection box, focussing on the polyp itself without background distractions. This approach allows the model to concentrate on feature analysis rather than polyp detection.

The models are evaluated using sensitivity, specificity, accuracy, and the area under the curve (AUC). Sensitivity assesses how effectively the network identifies positive cases, while specificity measures its ability to correctly classify negative cases. Accuracy reflects the overall correctness of the model’s predictions. The AUC provides a summary of the model’s performance across all classification thresholds by plotting true positive rates against false positive rates. These metrics are computed independently for each feature using the corresponding labels.

### Management of indistinguishable labels

This experiment investigates the impact of indistinguishable labels on training, where indistinguishable labels are either included as a third class or ignored by removing frames with at least one indistinguishable feature. The results, comparing model A trained without indistinguishable labels and model B trained with them, are presented in Table 5.

Both models are trained and tested only on a subset of the long procedures dataset. The two models exhibit very similar results, with differences of less than 1% for both sensitivity and specificity on all features except vessels. For vessels, the specificity is higher for the model trained without the indistinguishable label, with 91.9% against 84.1%. However, the sensitivity is consistently higher for model B. Considering the marginal performance differences ($\le$ 0.7%), it cannot be conclusively stated that model B is significantly superior. According to this experiment, the indistinguishable label does not significantly enhance overall results. Consequently, the final model is trained without the indistinguishable label, but the label is retained as a filter to remove low-quality frames from the data.

### Qualitative results

**Fig. 3:**
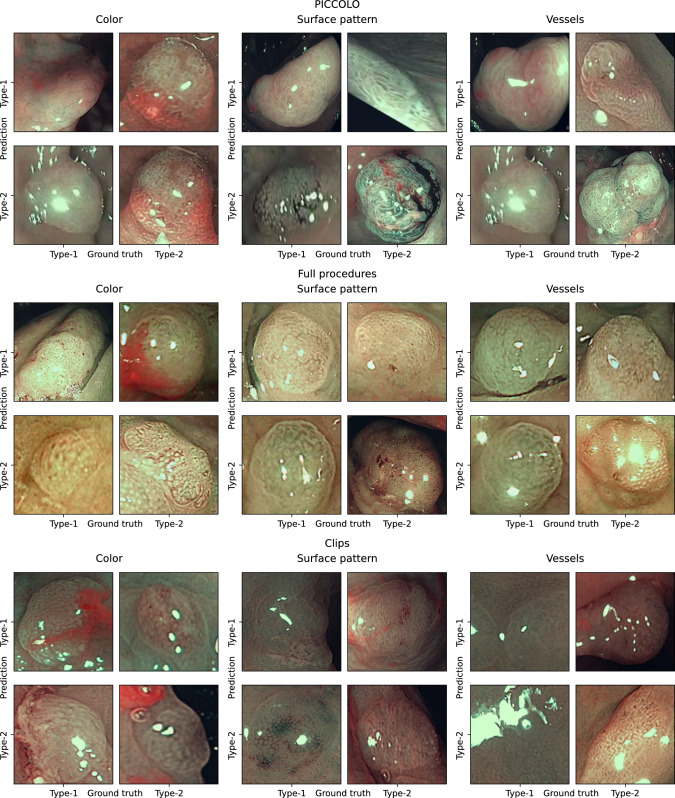
Examples of predictions from each dataset. The predictions are organized by each feature and displayed in a confusion matrix to compare the classification of type-1 and type-2 polyps

Examples of predictions from each dataset are shown in Fig. 3. These examples are provided before the thresholding step, meaning no rejections have been applied yet. It is important to note that some frames present significant challenges for feature classification, particularly due to light reflections. Additionally, distinguishing between type-1 and type-2 features can be difficult, as they sometimes appear quite similar. For instance, surface pattern and vessel classifications from the full procedures dataset illustrate challenging cases where the network struggled. While Fig. 3 highlights some of the most difficult examples and failure cases, it is reassuring that the majority of the data resembles the true positives and true negatives examples also depicted in the figure.

It is important to note that not all features are required to have the same classification type. For example, a type-2 polyp may exhibit type-1 colour or type-1 vessels. According to the official NICE classification, the presence of a single type-2 feature is sufficient to classify the polyp as type-2. In practice, however, these annotations are highly subjective and can vary between clinicians. In this study, features are evaluated independently, leaving their interpretation and combination for the final diagnosis to the clinician’s discretion.

**Table 6:** Results of the classification network on the three datasets for each feature before thresholding

	Accuracy (%)	Sensitivity (%)	Specificity (%)	AUC (%)
*Full procedures*				
Colour	90.6	92.6	86.0	96.8
Surface	89.2	93.2	80.8	96.4
Vessels	89.2	92.9	81.5	96.3
*Clips*				
Colour	88.1	87.1	90.1	95.2
Surface	88.1	87.7	88.8	94.6
Vessels	88.1	86.4	91.6	94.6
*PICCOLO*				
Colour	86.8	83.1	98.1	96.8
Surface	84.8	82.0	92.7	94.5
Vessels	84.9	80.3	99.0	96.4

Sensitivity evaluates the model’s ability to accurately classify type-2 features, while specificity assesses its effectiveness in correctly classifying type-1 features

**Table 7:** Results of the classification network on the three datasets for each feature after thresholding

	Accuracy (%)	Sensitivity (%)	Specificity (%)	AUC (%)
*Full procedures*				
Colour	95.0	95.8	93.1	98.5
Surface	94.4	96.8	88.4	98.3
Vessels	94.4	96.3	89.7	98.2
*Clips*				
Colour	92.2	90.9	95.0	96.8
Surface	92.3	91.7	93.6	95.8
Vessels	92.3	90.9	95.2	95.9
*PICCOLO*				
Colour	90.7	87.5	100.0	97.5
Surface	88.9	86.9	94.1	95.5
Vessels	89.2	85.4	100.0	97.1

Sensitivity evaluates the model’s ability to accurately classify type-2 features, while specificity assesses its effectiveness in correctly classifying type-1 features

### Quantitative results

The classification results for the features before applying the threshold are detailed in Table 6. The accuracies and AUC values are quite similar across different features within the same dataset, suggesting minimal differences in predictions between the features. All metrics reach at least 80%. The lowest values observed are the specificity of the surface features (80.8%) and vessel features (81.5%) in the full procedures, as well as the sensitivity in the PICCOLO dataset, with the surface features at 82.0% and vessel features at 80.3%. It is also worth noting that the colour feature consistently achieves high performance across all datasets, particularly in sensitivity, where it outperforms surface and vessel features in most cases. The specificity metric shows some variability, with the PICCOLO dataset displaying the highest specificity values, particularly for the vessels feature, which reaches 99.0%.

The results for the classification of polyp features after applying a threshold of 0.2 are shown in Table 7. The threshold was determined through an ablation study, representing the optimal balance between the number of rejections (fewer than 15% of the test set) and performance improvements. In the full procedures dataset, the colour feature achieves the highest accuracy at 95.0%. The surface and vessel features perform similarly, each with an accuracy of 94.4%, but they exhibit higher sensitivity (over 96%) and lower specificity (below 90%) compared to the colour feature. In the clips dataset, the three features yield very close results, all with accuracies exceeding 92%. The surface feature stands out with a slightly higher sensitivity of 91.7%, compared to 90.9% for the other features, but has a lower specificity of 93.6%, while the other features maintain specificity above 95%. In the PICCOLO dataset, the overall accuracy is lower, falling below 91% for all features. Specificity is notably high, reaching 100% for both colour and vessel features and 94.1% for surface. However, sensitivity decreases to below 88% across the board.

Compared to the results before thresholding, all metrics show improvement. Accuracy increases by 4 to 5%, while the AUC improves by 1 to 2% across all datasets and features. This enhancement is expected, as low-confidence predictions are discarded. The results indicate that the model rarely makes confident errors; instead, it tends to be uncertain when its predictions are incorrect, allowing those uncertain predictions to be excluded.

### Limitations

The primary limitation of this study is the lack of available data. The datasets used had to be manually labelled with NICE features, as no pre-annotated datasets exist. These annotations are inherently subjective, even when using the "indistinguishable" label. Additionally, the NICE classification system itself is not without flaws, leading to an unavoidable margin of error in the results. Therefore, the final decision should always rest with the clinician, who can consider all relevant factors, including the broader context of the video.

## Conclusion

In summary, this study introduces a robust model capable of classifying the three polyp features from the NICE classification with an accuracy exceeding 92% for internal datasets and surpassing 88% for the PICCOLO dataset. The proposed classification has the potential to support clinicians in making accurate diagnoses, providing a medically explainable approach. Notably, the study explored the influence of indistinguishable labels, finding that their inclusion did not significantly improve overall results. Future work aims to extend the model to integrate SSP using the Workgroup serrAted polypS and Polyposis (WASP) classification. This expansion is expected to enhance diagnostic capabilities, enabling the comprehensive handling of various polyp types. The study lays a strong foundation for further advancements in computer-aided diagnosis for colorectal cancer screenings.

## Data Availability

Data underlying the results presented in this paper are not publicly available at this time due to permissions in ethics collection.

## Code Availability

The code supporting the findings of this study is proprietary and not publicly available. It is currently being used in collaboration with industry partners and is subject to confidentiality agreements.
